# Whale Shark (*Rhincodon typus*) Seasonal Occurrence, Abundance and Demographic Structure in the Mid-Equatorial Atlantic Ocean

**DOI:** 10.1371/journal.pone.0164440

**Published:** 2016-10-26

**Authors:** Bruno C. L. Macena, Fábio H. V. Hazin

**Affiliations:** 1 Departamento de Pesca e Aquicultura, Universidade Federal Rural de Pernambuco, Recife, Pernambuco, Brazil; 2 Departamento de Oceanografia, Universidade Federal de Pernambuco, Recife, Pernambuco, Brazil; University of Illinois at Chicago, UNITED STATES

## Abstract

Whale sharks are generally associated with environmental factors that drive their movements to specific locations where food availability is high. Consequently, foraging is believed to be the main reason for the formation of whale shark aggregations. Feeding aggregations occur mainly in nearshore areas and are composed primarily of immature individuals. Conversely, aggregations of mature adults are rarely observed, and their occurrence is correlated with oceanic environments. Despite an increase in the number of whale shark studies, information on mating and parturition grounds is still lacking. In the present work, we assessed the ecological and behavioural aspects of the whale sharks that visit the archipelago of São Pedro and São Paulo (ASPSP), located ~1,000 km off the coast of Brazil in the equatorial Atlantic Ocean. Forty-nine whale sharks were recorded from February 2005 to May 2014. The estimated mean ± SD size was 8.27 ± 2.52 m (range: 2.5–14.0 m) with no significant differences in size across the year. The maturational stages were classified by size as immature (<8.0 m; 32.56%) and mature (>9.0 m; 46.51%); with almost half of the observed animals being mature specimens. The majority of sightings occurred between February and June. During this period, the ocean current weakens and the waters are enriched by eggs and larvae of fishes and invertebrates that attract marine life to forage. At the same time, evidence of reproductive activity in adult females (*i*.*e*. swollen abdomen and bite marks on the pectoral fins), and the potential mating behaviour exhibited by one male, suggest that the ASPSP area might also have a role in whale shark reproduction. Irrespective of its use for feeding or reproduction, this insular habitat serves as a meeting point for both juvenile and adult whale sharks, and may play an important ecological role for the species.

## Introduction

The whale shark *Rhincodon typus* (Smith 1828) is a pelagic and highly migratory filter-feeding species distributed around the globe in tropical and subtropical waters [1,2]. Past studies on the ecology and biology of whale sharks have suggested a relationship between their movements and environmental features, including sea surface temperature, chlorophyll *a* concentrations, bottom relief and ocean currents [3–12]. Commonly, environments with an optimal scenario for whale shark occurrence provide suitable conditions for primary and secondary productivity, as the main known purpose for whale shark aggregation is foraging [2,13–15]. Sites with predicted seasonal foraging aggregations of whale sharks offer the best opportunity to study the species on a regular basis. Information gathered from these phenomena, for instance, has been valuable to assess the seasonality of occurrence, aspects of population structure and dynamics of whale sharks in coastal waters of Australia [14,16]; the Gulf of Mexico [17]; the Gulf of California [18]; Belize [19]; the Seychelles [20]; and the Maldives [21].

In Brazil, the whale shark occurs in coastal waters from Ceará to Rio Grande do Sul States and at oceanic islands, like the archipelagos of Fernando de Noronha, São Pedro and São Paulo [22] and Trindade [23]. However, the knowledge of whale shark life history in Brazilian waters is still largely limited to the description of anecdotal sighting records, strandings and incidental catches [22, 24]. The only exception is in the archipelago of São Pedro and São Paulo (ASPSP) where data from sighting records have been systematically collected, and suggest a much higher frequency of occurrence from January to June, probably due to biological factors (*i*.*e*. food availability) [25].

ASPSP is a small and isolated oceanic archipelago located in the equatorial mid-Atlantic Ridge. Considered a hotspot for pelagic biodiversity [26] due to its strategic location in the middle of the Atlantic Ocean, the archipelago provides shelter for marine life and may serve as a stopover during large-scale migrations of pelagic species [27]. The archipelago is also an important feeding ground for commercially important pelagic fishes such as yellowfin tuna (*Thunnus albacares*), bigeye tuna (*T*. *obesus*), wahoo (*Acanthocybium solandri*), rainbow runner (*Elagatis bipinnulata*) and many species of sharks [28,29]. Most of these species gather at the archipelago between January and June, which coincides with the reproduction and high abundance period of flying fishes [30], and when the environmental conditions are suitable for reproduction and recruitment of fish larvae and invertebrates [31]. Consequently, Brazilian fishing boats have been operating in ASPSP since the 1980s [32,33]. Whale sharks, however, were never targeted by the fishery in the area, with no record of any specimen being ever caught. Classified as “endangered” by the International Union for Conservation of Nature (IUCN) [34], the whale shark is also protected by Brazilian law as an “endangered” species [35].

Whale shark aggregations known to date are generally size and sex segregated, with a predominance of immature individuals in coastal feeding aggregations [2]. Adult whale sharks are infrequently observed, and the majority of sightings have been recorded at oceanic locations, such as the Galapagos Islands [36], Baja California Sur [18,37], the Azores [12], St. Helena Island [38] and ASPSP ([25]; present study). However, complete information on the distribution of mature whale sharks, and on the location of mating and nursery grounds, if any, remains lacking, despite being crucial for the conservation of the species.

To help fill the gap of information on oceanic life history of whale sharks, trends of long-term sighting records in ASPSP were assessed to identify the seasonality of occurrence, relative abundance and population structure, with additional observations on habitat use and behaviour. The information provided here reinforces the hypothesis that oceanic habitats are crucial to whale shark life history, independent of age, and must be better understood to ensure the adoption of adequate conservation measures for both the sharks and this unique habitat.

## Material and Methods

The data used in this research was obtained with full approval of the Instituto Chico Mendes de Conservação da Biodiversidade of the Brazilian Ministry of the Environment (permit no. 14124–6).

### Study area

**Fig 1:**
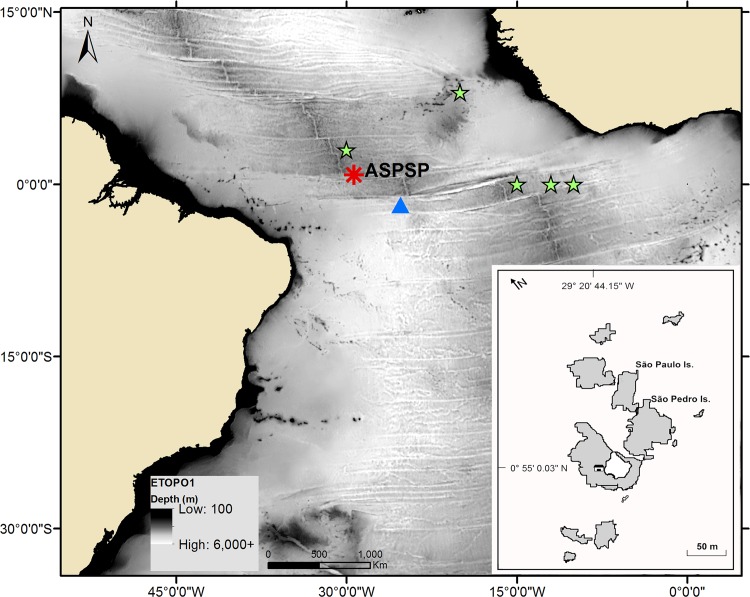
Study area. Geographical location and details (inset) of the Archipelago of São Pedro and São Paulo (ASPSP; red asterisk). The blue triangle indicates the satellite-tag pop up location from a female whale shark tagged in Caribbean Mexico [56]. The green stars indicate the location of recorded neonates [104,105].

The ASPSP is a remote group of small rocky islets, located in the mid-Atlantic Ridge, almost in the middle of the equatorial Atlantic Ocean (00°55’03”N; 029°20’45”W), approximately 100 km north of the equator and nearly midway between South America (1,100 km from Brazil) and Africa (1,600 km from Guinea Bissau) (Fig 1). The archipelago is part of an E-W seamount chain, located at the Saint Paul Transform Fault, rising from abyssal depths near 5,000 m, and presenting a rough bottom relief close to the islets [39].

The ASPSP region is directly influenced by the trade winds and by the Equatorial Current System, namely the South Equatorial Current (SEC) and the Equatorial Undercurrent (EUC), which control the dynamics of physicochemical and biological parameters around the archipelago. The intensification of the NE trade winds, between December and May, pushes the Intertropical Convergence Zone (ITCZ) southward, towards the archipelago [40], resulting in the wet season. During this period, the SE trade winds weaken, reducing the intensity of the westward flowing SEC in the area [41,42]. Conversely, during the dry season, from June to November, the ITCZ moves farther north of the archipelago due to the intensification of the SE trade winds [40], which strengthens the SEC [41,42]. The EUC, a very strong eastward subsurface current flowing at 50–100 m depth [43], is also directly influenced by the SE trade winds, becoming shallower and weaker between December and May and deeper and stronger from June to September [41,42].

### Data collection

#### Frequency of occurrence and abundance

The presence of whale sharks in the ASPSP was recorded by sighting surveys (SURV) through direct observation by on board or free diving observers, which were carried out during 37 scientific expeditions of 15 days each, from February 2005 to May 2014. Additionally, anecdotal sighting records were compiled (COMP) through interviews with local fishermen and other researchers, and collated with sightings from the literature [25] from February 2000 to May 2014. Sightings data included: date, time, location, number of individuals, estimated size and photographs, if available.

#### Oceanographic conditions

In order to characterize the oceanographic conditions in ASPSP surroundings, sea surface temperature (SST) (°C) remote sensing monthly image composites were obtained from the Advanced Very High Resolution Radiometer (AVHRR) aboard the NOAA Polar Operational Environmental Satellites (POES) (http://coastwatch.pfeg.noaa.gov/infog/AG_ssta_las.html). Chlorophyll-*a* concentration (CHL) (mg.m^-3^) remote sensing monthly image composites were obtained from the Moderate Resolution Imaging Spectroradiometer- MODIS-Aqua (http://oceancolor.gsfc.nasa.gov/). Remote sensing data were collected from January, 1, 2005, to December, 31, 2014.

#### Demographic structure

Whale shark total lengths (L_T_) (*i*.*e*. from the tip of snout to the end of the tail) were estimated to the nearest 0.5 m by comparing the size of the whale shark with known marks on a fishing vessel, after placing the boat in parallel with the shark, or with a diver of known size. Sex was determined in water by the presence or absence of claspers.

To assess the maturity of whale sharks visiting the ASPSP, shark sizes were compared to estimates available in the literature for other Atlantic Ocean sites. For males, information from the Mexican Caribbean [44] showed that 95% of males in that region were mature at 8.1 m. These authors, however, did not assess free-swimming female maturity, and no other information is available on female size at maturity in the Atlantic Ocean. Information from other ocean basins, suggests a size at maturity of 9.0+ m [2,45]. We thus chose, conservatively, to consider animals <8.0 m as juveniles, those >9.0 m as adults, and we did not classify animals within the potentially ambiguous 8.0–9.0 m size class.

#### Photo-identification

The area behind the fifth gill slit and in front of the first dorsal fin of the ASPSP whale sharks was photographed for individual identification and further population dynamic analysis [46,47]. Both left and right sides were photographed whenever possible. Additionally, information on other marks, scars, size and sex were also used to assist in identification. If available, images from collaborators (*i*.*e*. other researchers and fishermen) were also collected during the interviews and used for analysis if the quality was suitable.

### Data analysis

#### Comparison of frequency of occurrence between datasets

The relative frequency of occurrence (FO%) per month of whale sharks in the ASPSP was calculated by dividing the number of whale sharks sighted in each month by the total of whale sharks observed and multiplying by 100. A linear regression between the FO% per month was calculated using SURV and COMP data, and the similarity between estimations was assessed using the Welch *t-*test with the null hypothesis of no difference (H_0_ = true slope = 1) between datasets. The motivation for performing this analysis was to verify the reliability of the FO% of SURV when compared to the COMP for the period in which fewer scientific surveys were conducted.

#### Relative abundance index

Before the calculation of Sightings per Unit of Effort (SPUE), the data were filtered in order to minimize the potential duplicate sightings within each surveyed month. Since it was not possible to photo-identify all individuals to remove duplicates, the “short-term resightings” (*i*.*e*. recorded individuals with similar size and sex within a four day interval between sightings) were discarded. Although this does not entirely eliminate the possibility of inclusion of duplicates, it is expected to remove the majority of the multiple records (details in discussion). Relative abundance indices were calculated using only the SURV SPUE, expressed as the number of individuals sighted per day of expedition (sig.day^-1^), and grouped by median per month. The differences of SPUE between months and years were verified using Analysis of Variance (ANOVA) of one factor (month or year) with *post hoc* Tukey HSD, if differences were detected.

#### Oceanographic conditions

SST and CHL data with 0.1° and 0.05° of spatial resolution, respectively, were averaged in squares of 1° x 1° to characterize the general oceanographic conditions per month within a 100 km^2^ area around the ASPSP. The differences of monthly means of each variable were compared using ANOVA of one factor (*month*) with *post hoc* Tukey HSD test, if differences were detected.

#### Demographic structure

Size estimates of whale sharks using surface or underwater visual references tend to have an error of ± 0.5 m [14,19,48]. Since in the present work L_T_s were collected by both methods, the error of visual estimates was calculated based on the creation of a virtual random bias, standardized with fixed upper and lower constraints, and compared with the observed estimates in order to validate the SURV L_T_ for demographic analysis.

The error estimate of the SURV L_T_s based on the intervals of ±0.5 and ±1.0 m was generated, and considered as the bias in our visual estimations. A new dataset was then created, which randomly included three bias values (-0.5, 0.0, 0.5 or -1.0, 0.0, 1.0) to the SURV L_T_ to add the bias variance in the estimates. The mean was then calculated and a paired Student t-test was run to compare the mean of the new dataset with the SURV L_T_ mean. The process was looped 10,000 times to assure the use of all possible combinations of the three bias values; for each new random dataset generated, the mean and the p-value result from the t-tests were saved for further validation. Finally, we calculated the relative frequency of the number of t-test p-values which were smaller than 0.05 to assess if the bias assumed could be accepted. The validation was conditioned to the analysis of the quantity of p-value <0.05 which lies within the 95% confidence interval from all replicates. In other words, we generated 10,000 different datasets with standardized random bias, statistically compared each mean with the SURV L_T_ mean and verified the proportion of the t-test p-values <0.05 within the 95% of confidence interval to validate the SURV L_T_ visual size estimation.

The SURV and COMP L_T_’s were compared using Welch *t*-test and the mean size differences per month were compared using ANOVA of one factor (*month*) with *post hoc* Tukey HSD test, if necessary. The number of whale sharks in each maturity stage was compared to test the hypothesis of predominance of adults using Pearson’s chi square test. All statistical analysis were performed using the R programming environment v.3.2.2 [49].

#### Photo-identification

Photo-identification images were classified by their quality, processed following Speed et al [46], and analyzed using the I^3^S software [47]. The photo-ID dataset was composed of photographs from SURV and COMP. It should be noted that images from distinct occasions could represent the same individual if only one side were photographed [14].

The left side was chosen for analysis since there were more left side images, and because this is the standard established for the online whale shark global database “Wildbook for Whale Sharks” at www.whaleshark.org. Some whale sharks had both sides photographed and we were able to compare these with the sharks which had only the right side. All photo-identified individuals were compared within our own database and also submitted to the “Wildbook for Whale Sharks” to compare with images of other individuals identified around the world. When only one of the flanks was recorded, to assist the visual confirmation of identification analysis and to avoid duplicity in the photo-identification, more than one character (*i*.*e*. scars and stripe patterns) were used in parallel with individual intrinsic characteristics such as size and sex.

## Results

### Frequency of occurrence and relative abundance index

Forty-nine whale sharks were sighted between March 2005 and May 2014 over 555 expedition days (SURV), whereas 92 sightings were compiled from February 2000 to May 2014 (COMP), resulting in 141 combined sightings. Eighteen whale sharks, 5 from SURV and 13 from COMP, were excluded from the analysis by the filtering procedure.

**Fig 2:**
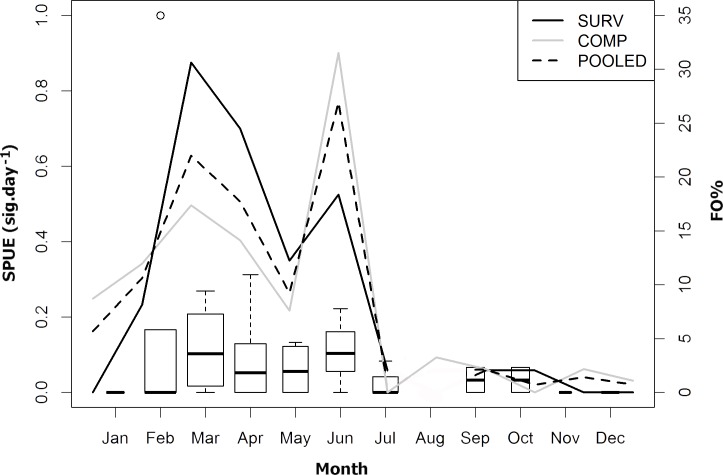
Distribution of whale shark sightings per month. Relative frequency of occurrence (FO%) per month of the SURV (n = 49; solid black), COMP (n = 92; solid grey) and POOLED (n = 141; dashed black) datasets; and SURV Sightings per Unit of Effort (SPUE; sig.day^-1^) per month of *R*. *typus* in the ASPSP. The width of the boxes is proportional to the square-roots of the number of observations in the groups, the horizontal bar is the median and the open circle indicates a single outlier.

FO% trends of the independent datasets, SURV and COMP, were similar, presenting a related sighting distribution, with peaks in the same months (March and June), but with an intriguing decrease in May (Fig 2). The comparison of COMP with SURV FO% revealed positive correlation between datasets (r^2^ = 0.611; p = 0.002), with the linear model slope (β_1_ = 0.93) very close to the null hypothesis (H_0_ = true slope = 1) and no statistical difference detected (Welch: t = 0.019, df = 21.388, p = 0.984). It was considered therefore, that the COMP fulfilled its purpose, which was to supplement the SURV with information on sightings records during the months when the research team was not in the ASPSP, particularly during the last six months of the year (Fig 2).

The SPUE dataset had 43 sample units (*i*.*e*. months). The months with highest median SPUE were June (0.1034) and March (0.1031), followed by May (0.0566) and April (0.0556; Fig 2). No expedition was conducted in August. Differences were not detected among the months SPUE (ANOVA: F = 0.433, df = 11, p = 0.929). The independent FO% of COMP was also consistent with the SPUE and presented trends of increasing abundance between February and June with peaks in March and June and decrease in the last six months (Fig 2).

**Fig 3:**
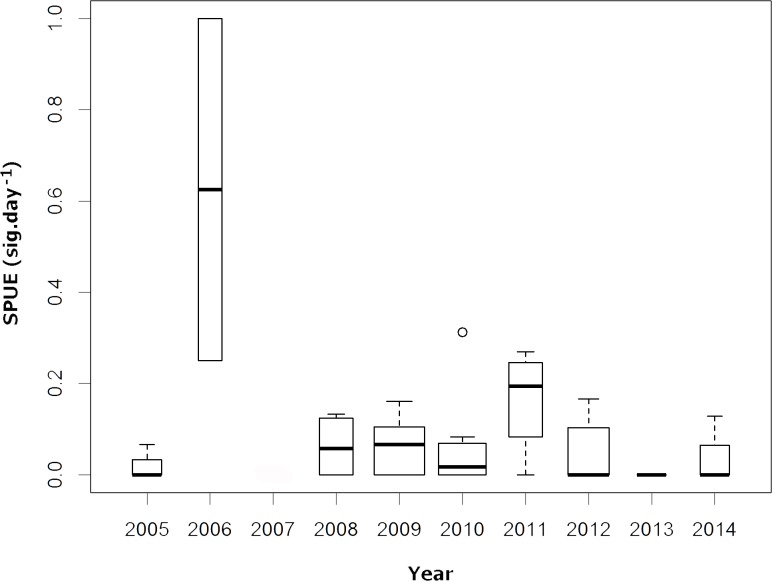
Distribution of whale shark sightings per year. SURV Sightings per Unit of Effort (SPUE; sig.day^-1^) per year of *R*. *typus* in the ASPSP. The width of the boxes is proportional to the square-roots of the number of observations in the groups, the horizontal bar is the median and the open circle indicates a single outlier.

The median SPUE per year spanned from nearly 0 in 2005, 2012, 2013 and 2014 to 0.625 in 2006 (Fig 3). No expedition was conducted in 2007. Statistically significant differences were only found between 2006 and all other years (ANOVA: F = 5.082, df = 9; p = 0.000248; TukeyHSD: p < 0.001). The high SPUE observed in 2006 was due to an expedition undertaken between February and March of 2006, when three whale sharks were sighted in the three days of the expedition that fell during February, thus generating an SPUE = 1 (Figs 2 and 3).

### Oceanographic conditions

**Fig 4:**
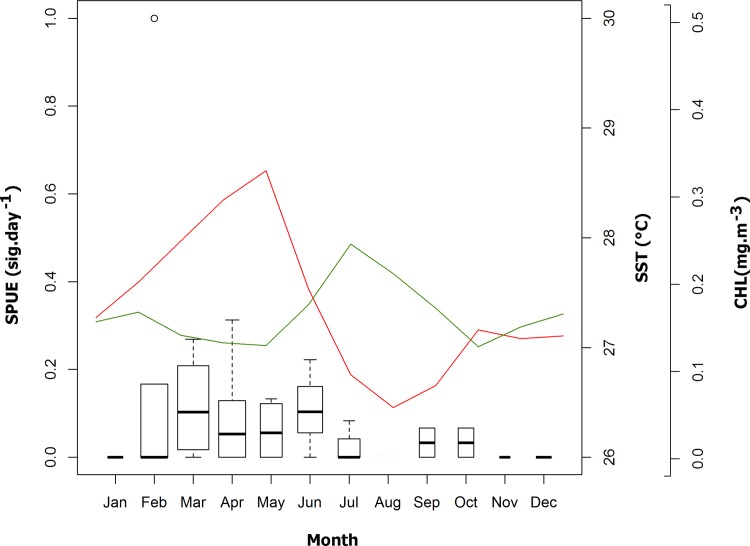
Primary productivity and sea surface temperature of ASPSP surroundings. Sighting per Unit of Effort (SPUE) in relation to sea surface temperature (SST; red line) and chlorophyll *a* concentration (CHL; green line) satellite image composites, from January 2005 to December 2014 for a ~100 km^2^ area around the ASPSP. The width of the boxes is proportional to the square-roots of the number of observations in the groups, the horizontal bar is the median and the open circle indicates a single outlier.

The SST increased gradually from August until a peak in May, subsequently decreasing from June to August (Fig 4, red line). Significant differences in SST were found (ANOVA: F = 2785, df = 11, p = <0.001) among almost all the months (TukeyHSD: p < 0.05; S1 Table). The CHL concentration was lower in October, slightly increasing from November to February, further decreasing between March and May, and finally increasing from June to July (Fig 4, green line). Differences in CHL were found (ANOVA: F = 403, df = 11, p = <0.001) among almost all the months (TukeyHSD: p < 0.05; S1 Table). The whale shark SPUE in relation to the oceanographic variables showed SST and CHL preferences ranging from 27 to 29°C and 0.10 to 0.16 mg.m^-3^, respectively (Fig 4).

### Demographic size structure

**Fig 5:**
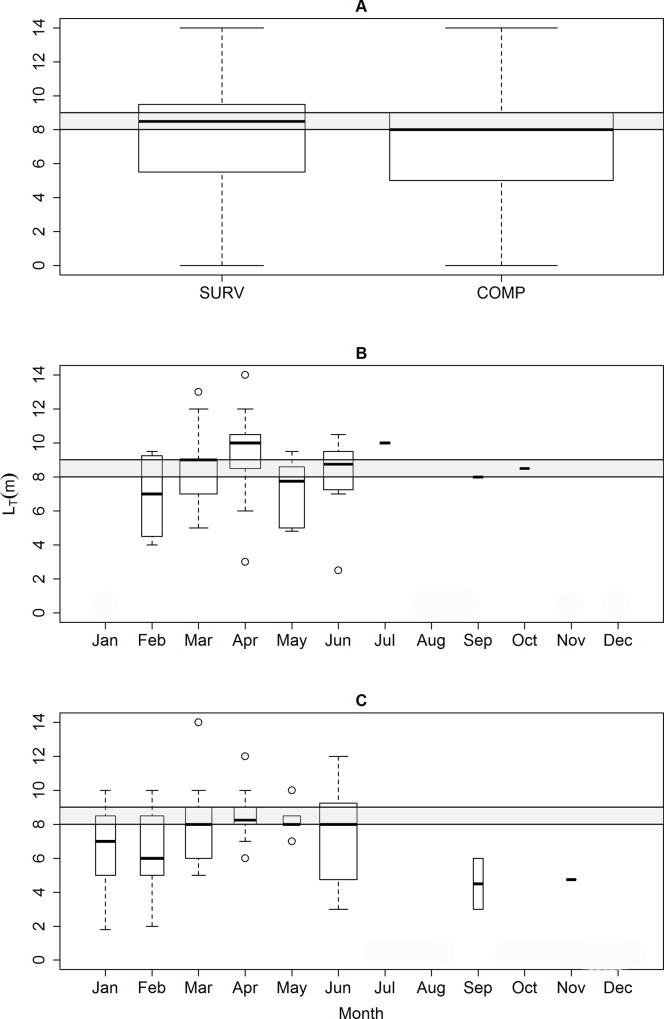
Distribution of whale shark lengths. (A) Comparison of *R*. *typus* sizes between SURV and COMP; and (B,C) size distribution per month for (B) SURV (n = 43) and (C) COMP (n = 79) in the ASPSP. Immature (below) and mature (above) animals are separated by a shaded area indicating the transitional 8.0–9.0 m size class. The width of the boxes is proportional to the square-roots of the number of observations in the groups, the horizontal bar is the median and the open circles indicate outliers.

The mean ± SD size of whale sharks recorded in SURV was 8.27 ± 2.52 m (range: 2.5 to 14.0 m; n = 43) and was statistically different (Welch: t = 2.167; df = 83.28; p = 0.033) from COMP (7.24 ± 2.44 m; range: 1.8 to 14.0 m; n = 79) (Fig 5A). Despite the overall mean difference of 1.02 m, the changes in SURV and COMP sizes throughout the year was quite similar (Fig 5B and 5C). Given the difference between the size estimates, we decided to perform demographic analysis only with the SURV dataset. The senior author made 86.4% of the SURV size estimates, while the remaining SURV observations were done by one other biologist.

Demographic analysis of whale shark sizes was conditioned to the calculation of the error in the size bias to validate the visual estimates. The L_T_s from the resampled analysis (bootstrapped) resulted in 10,000 dataset replications producing an equivalent number of means and p-values from the t-tests performed in each run. The mean of the resampled dataset for ±0.5 bias was 8.26 m, spanning from 7.94 to 8.59 m. The proportion of resampled dataset means which had significant differences (*i*.*e*. p<0.05) was 4.41%, within the confidence interval of 95% indicating no difference between the SURV L_T_ and the resampled means. Thus the bias in visual estimate was considered acceptable and used for further demographic analysis. Comparable results were obtained using a bias of ±1.0 m, where only 4.99% of the replicates had significant differences with an overall mean of 8.27 m spanning 7.87 to 8.68 m. Despite the slight increase in whale shark mean L_T_ observed from February to April (Fig 5B), these differences were not statistically significant (ANOVA: F = 0.547, df = 7, p = 0.793).

**Fig 6:**
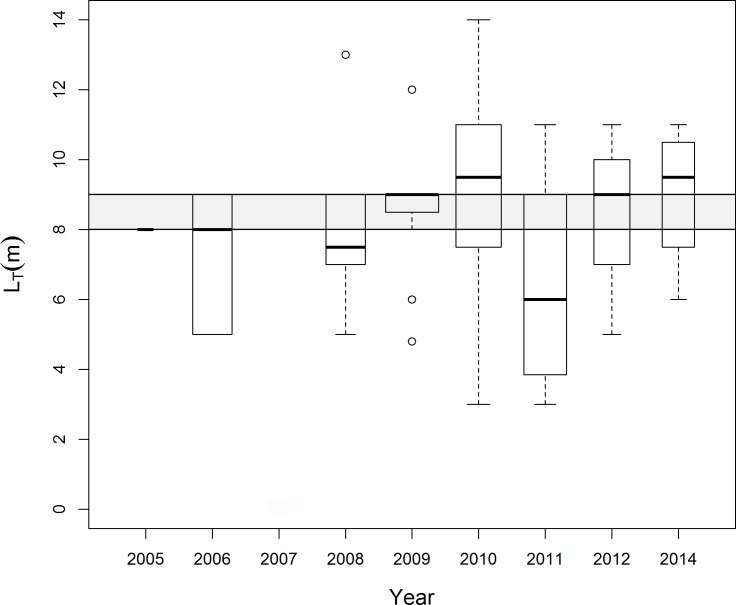
Annual distribution of whale shark lengths. Size distribution per year (SURV; n = 43) in the ASPSP. Immature (below) and mature (above) animals are separated by the shaded area indicating the transitional 8.0–9.0 m size class. The width of the boxes is proportional to the square-roots of the number of observations in the groups, the horizontal bar is the median and the open circles indicate outliers.

In almost all years the mean L_T_ was equal or above 8.0 m; the two exceptions, 2006 and 2011, had means of 7.3 and 6.5 m, respectively (Fig 6). The years with largest and smallest means were 2010 (9.1 m) and 2011 (6.5 m) but no difference in sizes were found between the years (ANOVA: F = 0.75, df = 7, p = 0.632).

**Fig 7:**
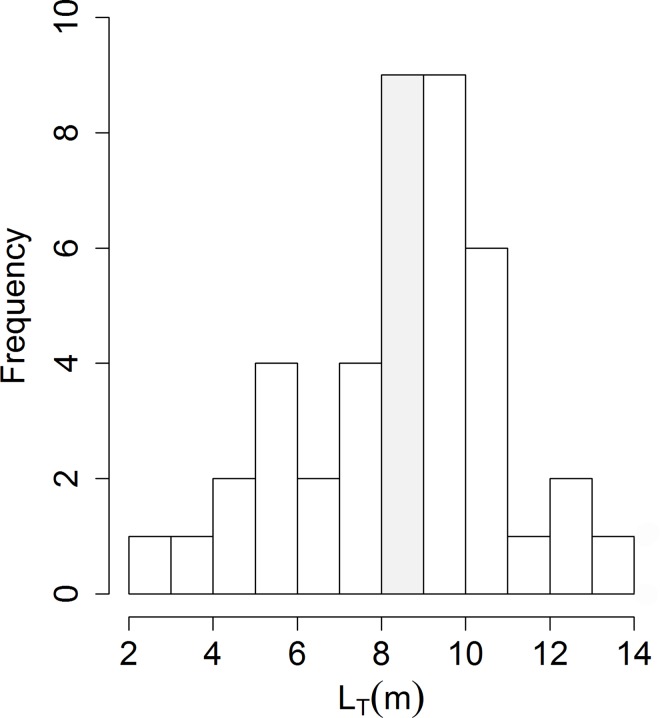
Length frequency of whale sharks. Absolute frequency of *R*. *typus* L_T_ (m) in the ASPSP. Immature (left) and mature (right) are separated by the shaded area of transitional size animals.

The size frequency distribution exhibited a continuous distribution from 8.0 to 10.0 m with a minor peak at 10.0–11.0 m (Fig 7). The mean ± SD of immature and mature sharks were 5.37 ± 1.53 m and 10.25 ± 1.46 m, respectively. Based on the estimated size, 32.6% (14) of sharks were immature, and 46.5% (20) were mature. The remaining 20.9% (9) belonged to the 8.0 to 9.0 m class, not included in the demographic analysis. No differences in number of individuals were found between the two maturity classes (Pearson’s χ^2^ = 1.058, df = 1, p-value = 0.303). The sex was identified in 14 records (28.6%; n = 49), of which 11 were females (78.6%) and 3 were males (21.4%), a sex ratio of 3.7:1, with mean L_T_ ± SD of 9.5 ± 1.3 m (range: 6.0 to 12.0 m) and 9.4 ± 4.6 m (4.7 to 14.0 m), respectively.

Of 768 photos and 133 videos (118.58 min), 27 whale sharks were recorded in the identification area, although after quality inspection only 16 had images considered adequate for photo-identification. I^3^S software was used to analyze the spot patterns from these 16 animals, nine sharks with photos from the left or both sides and seven animals with photos only from the right side, which were compared only with sharks that had images from both sides. One whale shark was identified only by a remarkable scar (absence of first dorsal fin). Only two re-sights were found among the 16 (36.4%; n = 49) whale sharks photo-identified in the ASPSP. These two identifications (12.5%, n = 16) were a 10.0 m female and a 5.5 m male re-sighted one and three days after the first encounter, respectively. No match for any of the ASPSP sharks was found in the “Wildbook for Whale Sharks” global database.

**Fig 8:**
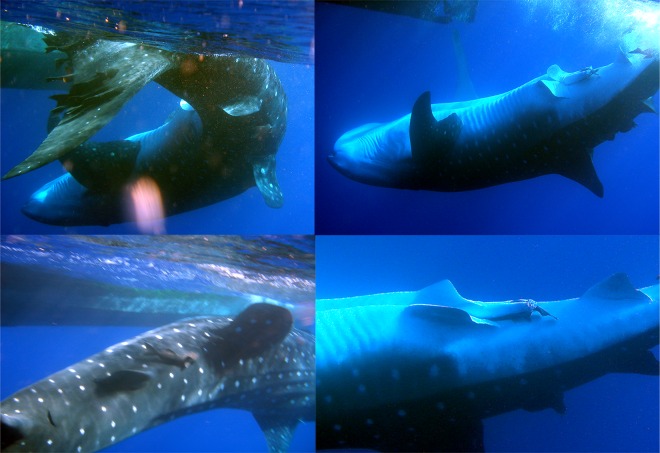
Male potential courtship behaviour. Multiple rolling behaviour by a large male *R*. *typus* displayed with the fishing vessel and close up of its clasper abrasion recorded in the ASPSP. Credit: Sibele Mendonça©.

**Fig 9:**
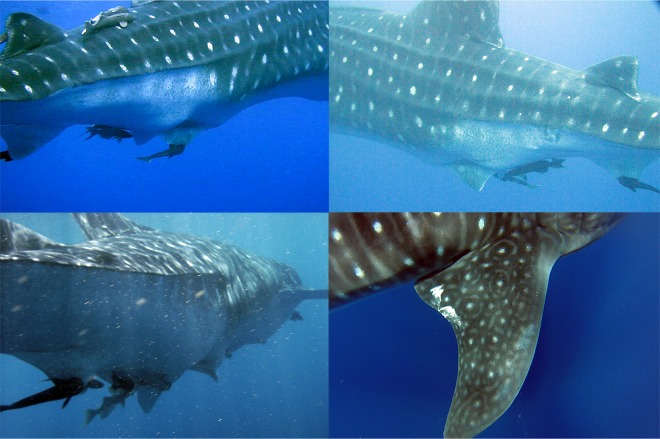
Females showing indications of reproductive activity. *R*. *typus* females showing swollen pelvic region and bite scar on the pectoral fins, suggestive of mating, recorded in the ASPSP. Credit: Bruno Macena©.

Generally the whale sharks seen in the ASPSP exhibited solitary behavior. Only seven conspecific associations were recorded, with three individuals observed in the same moment on two different occasions. All other associations were composed of two sharks. An adult male with an apparent abrasion of the claspers displayed an atypical behaviour of repeatedly rolling the body longitudinally alongside and below the fishing vessel (Fig 8), diving and returning near the boat three times within a 10 min interval. Furthermore, some females presented a distinctly swollen pelvic region and one female had scars on both pectoral fins (Fig 9). Both of these findings may be suggestive of reproductive behavior.

Only on five occasions were whale sharks observed feeding by the research team; three times during the day and twice at night. Fishermen also reported several night foraging events near the boats. Surface vertical and ram-filter feeding behaviours were observed during feeding activities during both day and night. In several instances, other marine organisms were seen alongside whale sharks. The most commonly observed were remoras (*Remora brachyptera*, *R*. *osteochir*, *R*. *remora*, *Remorina albescens*), which were attached to the sharks in large numbers (up to 23 on one individual), and Chilean devil rays (*Mobula tarapacana*). Other species recorded were rainbow runner (*Elagatis bipinnulata*), almaco jack (*Seriola rivoliana*), blackjack (*Caranx lugubris*), ocean sun-fish (*Mola mola*), pilot fish (*Naucrates ductor*), yellowfin tuna (*Thunnus albacares*), blackfin tuna (*T*. *atlanticus*), silky shark (*Carcharhinus falciformis*), scalloped hammerhead shark (*Sphyrna lewini*), bentfin devil ray (*M*. *thurstoni*), and bottlenose dolphin (*Tursiops truncatus*).

## Discussion

### Frequency of occurrence and relative abundance

Information collected through collaborators (*i*.*e*. diving operators, fishers, citizens), and compiled in datasets analogous to direct science-based surveys, have been used in scientific research to increase the capacity of data acquisition or to promote data collection in areas which require complex logistics [50–52]. Although traditional approaches used in scientific studies involve rigorous standardized techniques based on tested and approved methods [53], such parallel databases have intrinsic limitations (*i*.*e*. biases) that prevent their use for direct inferences for abundance, biomass and density of shark populations [53,54]. Scientific survey data combined with citizen-science data, however, have been successfully used to identify seasonal frequency of occurrence and population structure and dynamics of whale sharks in Australia [14,16,51,55]. Nevertheless, in order to use an analogous dataset in a reliable manner, an independent *in situ* validation is necessary [52]. In the present case, the reliability of the COMP FO% from the collation of the SURV dataset was successfully verified.

Logistic difficulties can affect the development of research programs at remote and inhospitable islands such as ASPSP. Since previous research [25] and records from fishermen had already indicated that whale shark FO% in the area was much higher during the first six months of the year, from 2009 on the major part of the research effort was carried out during this period to facilitate the deployment of satellite tags and the collection of tissue samples.

### Seasonality

Whale sharks are widely distributed in the Atlantic Ocean, occurring in Central America (Caribbean Sea and Gulf of Mexico (GOM) [56]), Northwest Atlantic [57]; Northeast Atlantic [12], Equatorial Atlantic [25], Southeast Atlantic [58] and Southwest Atlantic ([22]; BCLM, unpub. data). In all these locations, the whale sharks appear to show distinct spatio-temporal distributions. Strategically located in the middle of the Atlantic Ocean, the ASPSP may play an important role in the transoceanic cycle of the whale shark in the Atlantic, as suggested by a satellite-tagged female that moved from the GOM towards the mid-Atlantic Ridge, with the tag popping-off near ASPSP [56] (Fig 1). However, other evidence of connectivity between Atlantic Ocean locations, from satellite tracking or photo-identification, have so far only been found in Central America [56].

In the Galapagos, of 82 individual whale sharks photo-identified, only 12 sharks were re-sighted within a 7-day period, and only one shark was re-sighted between years [36]. The same authors found no matches when searching for intra and inter-annual re-sightings in the “Wildbook for Whale Sharks”. These results are quite dissimilar from the coastal aggregations where the re-sighting rates are high, with intra and inter-annual matches detected and residence time varying from 11 to 180 days [37,48,56,59].

Our photo-identification analysis detected only two re-sightings with a short period of time between the encounters. Given the lack of re-sightings it was not possible to apply demographic models, thus preventing any inference regarding population size, residency time or fidelity. The absence of long-term re-sightings in the area may be explained by (1) the reduced photo-ID sample size, if the whale sharks do return to the archipelago but were not re-sighted or (2) absence of return on a long term basis. The short residence time (~2 days), strong intra-seasonal abundance and high turnover rate of Galapagos whale sharks [36] helped to define the assumptions of the SPUE filtering technique used here to avoid duplicates, considering the similarities between the Galapagos and ASPSP habitats. To explain the trends of occurrence of whale sharks, it is necessary to understand the dynamics of oceanographic, atmospheric and biological phenomena in the area.

Araújo and Cintra [60] used hypothetical models of particle dispersion to predict larval plankton retention/recruitment, and ocean circulation to identify potential increases in primary productivity in the ASPSP. The authors estimated a higher probability of larval retention/recruitment in February (SEC with lowest zonal speed), whereas in June (SEC with highest zonal speed), the inverse was observed. The ocean circulation models indicated small areas of potential submerged topographic upwelling at the east side of the ASPSP, between 100–150 m depth. The latter conclusion is probably a consequence of the strengthening of the EUC, as a result of the interaction between this subsurface current and the rough bottom relief of the ASPSP area [39,61]. Nevertheless, no large-scale upwellings have yet been described in the ASPSP area [62,63]. A small scale, seasonal sea-water enrichment, however, is observed during the rainy season (February to May); when the increased precipitation caused by the ITCZ results in a runoff of nutrients from excretion of the abundant marine birds that congregate at the ASPSP (BCLM, *pers*. *obs*.).

### Opportunistic feeding ground hypothesis

The whale shark swims independently of the ocean currents [7]; but ocean currents may provide clues on potential feeding opportunities, therefore influencing the movement of fishes [5,6,64,65]. The filter-feeding whale shark feeds mainly on invertebrate and/or fish spawn and larvae, squid and schooling fishes (reviewed in [2]). They aggregate to feed in specific seasons and locales where oceanographic (*i*.*e*. upwelling) or biological (*i*.*e*. fish or invertebrate spawning) phenomena occur [13,15,17,66]. In the Coral Sea, during the lantern fish spawning period, whale sharks associate with tuna to forage [67]. The association of whale sharks with tuna is observed elsewhere [9,12,44,68], and they may commonly forage on the same prey.

Yellowfin tuna and wahoo (the two main species fished in the ASPSP) were also the most abundant species caught during the first six months of the year [33]. Both species, as well as other fishes and sharks, prey on flying fish [28,29], the third most important fishery resource in the ASPSP [33]. In ASPSP, records of whale shark feeding behaviours (description in [15,18,69] are rare, but observed on some occasions. Fishermen from ASPSP reported several foraging events in which whale sharks preyed on flying fish during the night (or their eggs and larvae), but no large feeding aggregation was observed. Therefore, despite the lack of large foraging events recorded in ASPSP, the largest concentration of planktonic organisms observed in the first six months of the year coincides with the highest abundance of whale sharks. Whale sharks may therefore use of the ASPSP area as a feeding station during their oceanic migration.

The timing of whale shark sightings in ASPSP coincides with the period of lowest current speed, highest SST and lowest CHL values. The later may suggest a potential lagged response between whale shark presence and CHL, as observed in the Azores [12] and India [8], since they actively prey on zooplankton and small planktivorous fishes. Although the strengthened oceanic currents from June to August probably increase the levels of CHL due to higher nutrient contents in the water resulting from submerged upwellings in response to the rough topography, they would also tend to carry zooplankton organisms away from the archipelago. On the other hand, the CHL increase from November to February may be responsible for the biological enrichment of the waters around ASPSP from February to May, as the ocean currents are weakening and the larval retention/ recruitment is higher during this period.

The ASPSP offers optimal conditions for reproduction, spawning, larval development and feeding of invertebrates and fishes [31]. Water temperature is known to induce fish reproduction/spawning events [70–72]. Fish reproduction studies conducted in ASPSP have indicated spawning periods of several species mainly between January and June [30,73–76]. The abundant sally lightfoot crab (*Grapsus grapsus*) reproduces in ASPSP during the whole year, but largest abundances of ovigerous females were observed from December to May [77]. The zooplankton near ASPSP was dominated by copepods followed by brachyuran crab larvae (zoea), with higher density during the night and in warmer months [78,79]. Additionally, the most abundant fish larvae in ASPSP were the flying fishes (Exocetidae), halfbeaks (Hemiramphidae) and lantern fishes (Myctophidae) with the highest abundances increasing with distance from the archipelago [30,31]. This could explain why whale shark foraging events were not seen with greater frequency during the day and closer to the ASPSP, where the majority of the surveys were carried out.

### Demographic structure

Sexual and ontogenetic segregation is common in shark species [80,81], including whale sharks [2]. Most coastal whale shark aggregations are composed predominantly of immature males, at sites such as Western Australia [14,55], Djibouti [82], Seychelles [5], Philippines [83], Maldives [21], Belize [19], Honduras [59] and Mexico [17,37,44]. While both large (>9 m), and female, whale sharks are seen less frequently in these aggregations, they are commonly observed at oceanic sites such as in in the Azores [12], at Saint Helena [38], at Baja California Sur [18,37], at the Galapagos Islands [36], and as we show here at the ASPSP. In Baja California and the Galapagos, a great number of adult females were observed, including potentially gravid ones, as inferred by their distended pelvic region. Nevertheless, the only confirmed pregnant female recorded to date was caught in Taiwan [84]. The size of ASPSP whale sharks ranged from 2.5 m to 14.0 m (mean = 8.27 m) with roughly equal numbers of immature and mature animals, indicating an absence of ontogenetic segregation. This type of structure is uncommon worldwide, as most other sites show primarily immature or mature animals, but not both. Similar size distributions have been observed in Taiwan and India, with whale sharks spanning from 1.0 to 13.0 m (mean = 4.6 m) [85] and from 3.1 m to 14.5 m (mean = ~7.0 m) [86], respectively, but in both locations the number of immature animals was considerably greater than that seen at ASPSP.

In the Galapagos, the whale sharks have been found to span from 4.0 to 13.1 m with large females dominating (91.5%), and the mean size of immature (5.33 m) and mature (11.35 m) [36], close to that observed at ASPSP. Similarities between the Galapagos and ASPSP are significant, as both are isolated oceanic environments located in the equatorial region. Given the similarity of the habitat, it is perhaps not surprising they have a similar population structure composed of transient adult females with a high incidence of pregnancy. In St. Helena, preliminary results show an equal mix of mature male and females with sizes varying from 8.5 to 11.0 m in length. Additionally, the authors suggest evidence of mating behaviour in the area based on two anecdotal records [38]. Given the information on demographic structure of whale sharks in pelagic environments, oceanic habitats appear to have important roles in the reproductive cycle of whale sharks.

The age at sexual maturity of a given species is a critical factor in evaluating the dynamics of its population, particularly for endangered or vulnerable species, and for those with slow maturation rates [34,87]. In Western Australia, Norman and Stevens [55], indicated that ~10% of male whale sharks less than 8.0 m were mature, based on clasper morphology, while 50% and 95% were adult at 8.1 m and 9.1 m, respectively. About 50% of male whale sharks from South Africa and Mozambique (also Indian Ocean sites) were mature at 9.1 m [88]. In the Mexican Caribbean (Atlantic Ocean), 50% and 95% of the males were mature at 7.0 m and 8.1 m, respectively, based on clasper morphology [44]. Female sharks commonly reach maturity at larger sizes than males. Whale shark females smaller than 9.0 m dissected in India [89,90] and South Africa [91] were all immature, while the smallest mature female observed in Taiwan was 9.6 m [92]. Geographic differences in size at maturity have been observed in other shark species [93–95] and may also occur for whale sharks given the information above.

Size estimates of whale sharks, using surface reference or underwater visual observation, tend to have an error of ± 0.5 m [14,19,48], particularly if the sharks are >8.0 m [36,96]. Visual measurements of whale sharks compared with laser photogrammetry resulted in calculated errors of *c*. ± 0.70 m and less than one meter, in Mozambique [97] and Galapagos [36], respectively, of visual estimations. In Western Australia, the visual estimate error was calculated between 0.75 and 1.49 m compared with stereo-video camera [96]. The studies compared above had a tendency to underestimate the visual measurements of the sharks compared to the more reliable measurements techniques. Considering these errors of visual estimation, the bias of ±0.5 and ±1.0 m used to validate our visual measurements seemed reasonable, thus the SURV L_T_ data was used to perform demographic analysis. Given the differing estimates of whale shark size at maturity noted in the literature, and the potential for 0.5 to 1.0 m error in size estimation, we chose to exclude the 8.0–9.0 m transitional size category from the demographic analysis.

### Reproductive ground hypothesis

Despite the absence of a statistically significant difference in monthly mean sizes of whale sharks at the ASPSP, a slight increase between February and April is noticeable and may suggest that the largest specimens are arriving in the area during the peak period of abundance. Movement of satellite-tracked whale sharks from the Gulf of Mexico revealed that a 7.5 m female, with external evidence of possible pregnancy, traveled from Holbox, Mexico, through the mid-Atlantic Ocean [56]. The tracking started in August and stopped after the tag detached in January, at a position 543 km southeast from the ASPSP (Fig 1). The location and timing of tag detachment coincides with the beginning of the warmest period in the equatorial region. There is evidence for reproductive behavior at the ASPSP in other elasmobranch species as well. In devil rays (*M*. *thustoni* and *M*. *tarapacana*) in the ASPSP, reproductive behaviour (following, close swim, and grouping) and anatomical evidence of mating (bite scars in females and abraded claspers in males) have been seen in captured animals and by underwater recordings between March and June in ASPSP (BCLM, *pers*. *obs*.). Additionally, one early pregnant *M*. *thurstoni* [98] and a mid-term pregnant scalloped hammerhead shark (BCLM, *pers*. *obs*.) were captured in March and April, respectively, indicating the use of ASPSP as part of the reproductive cycle for some elasmobranch species in the first six months of the year. Whale sharks of 8.0 to 9.0 m, that are completing their maturation and moving into their reproductive lifespan, may be making use of the warmer waters of the equatorial Atlantic and of the higher food abundance in the ASPSP, compared to the oligotrophic open ocean surrounding it.

The mean sizes of both male and female whale sharks seen at the ASPSP were close to that of mature animals. In April 2010, a solitary male was seen performing what appeared to be a mating behavior, swimming very close to the boat, rolling longitudinally and curving the body ventrally three times within a 10 min interval. This male showed abrasion of the claspers (Fig 8) indicating mating activity may have occurred recently [55]. In Seychelles, a 9.5 m whale shark was videoed performing exactly the same behaviour in relation to the research boat (D. Rowat, *pers*. *comm*.) Martin [99] noted putative courtship behaviours of following and parallel swimming performed by whale sharks in Western Australia. Many reproductive behaviours of elasmobranchs have been already described [100] but the longitudinal rolling observed in ASPSP and Seychelles appears to be a new behavior.

The lack of neonates and/or large females in coastal aggregations suggests that mating/pupping areas of whale sharks are likely to be located far from the coastal environment. Conversely, the presence of gravid females in oceanic regions, such as Baja California Sur [18,37] and Galapagos Islands [36], St Helena [38] and now the ASPSP, and the concurrent occurrence of small juveniles in areas from major ocean basins such as Indian (Djibouti [82]; India, Pakistan, Bangladesh and Seychelles [101]); Indo-Pacific (Philippines [102]; Taiwan [103]); Pacific (open ocean [104]) and Atlantic (equatorial open ocean [104,105]; ASPSP ([25]; present study), provides clues to where reproductive activity may occur worldwide.

These data seem to support the hypothesis that whale shark mating and/or parturition might occur in the deep ocean [46,56,82,99], and oceanic features (*i*.*e*. seamounts and islands) like ASPSP may offer suitable conditions for the development of part of the reproductive cycle of this species. Despite the lack of additional indicators of reproductive activity of whale sharks in ASPSP, due to the difficulty of direct *in situ* observation, the evidence from the animals presented here raises the possibility that whale sharks use the ASPSP for reproductive purposes. The suspected gravid females plus the young whale sharks observed in ASPSP, combined with recorded neonates in the equatorial Atlantic Ocean ([104,105]; Fig 1), suggest that the surrounding areas of ASPSP could be also used as pupping ground. Additional evidence and/or the development of new techniques that allow the identification of sexual maturity of free swimming sharks are needed to better understand the reproductive ecology of whale sharks and the role of this remote archipelago. The use of satellite tags at ASPSP may help to elucidate the migration patterns of young sharks and potential pregnant females in the Atlantic Ocean.

## Conclusions

The majority of the studies on whale sharks have been carried out on coastal feeding aggregations, with few studies developed so far in deep-water oceanic regions. Information on where whale sharks reproduce (*i*.*e*. mating and pupping areas) is crucial to the development of appropriate conservation measures at regional and international levels. The present study provides information on the ecology and biology of whale sharks visiting an isolated oceanic habitat located in the middle of the equatorial Atlantic Ocean, the ASPSP. The seasonality of occurrence is likely related to the oceanographic and biological features of the area, suggesting that whale sharks could be using the ASPSP to opportunistically feed during their transoceanic migration. The demographic structure of the ASPSP aggregation is quite different from most other aggregations, with a lack of size segregation resulting in individuals ranging from small juveniles to large adults. Juvenile and adult whale sharks may be using the archipelago with different purposes. Reproductive indicators suggest that the archipelago could serve as a mating and/or pupping ground, although more information is needed to test these hypotheses. Regardless of its role, the ASPSP insular habitat is important from an ecological point of view and represents a unique opportunity to gather relevant information on this iconic species.

## Supporting Information

S1 Table Summary of TukeyHSD test results from the comparisons environmental variables per month. Output from TukeyHSD test of monthly comparison of sea surface temperature (SST) and chlorophyll a concentration (CHL). Bold values indicate no significant difference (p > 0.05).
(DOCX)
