# Supplementary material for: Whale Shark (Rhincodon typus) Seasonal Occurrence, Abundance and Demographic Structure in the Mid-Equatorial Atlantic Ocean
Source: PLoS One. 2016 Oct 26;11(10):e0164440. doi: 10.1371/journal.pone.0164440 (PMC5082610; doi:10.1371/journal.pone.0164440)
Supplement: S1 Table — Output from TukeyHSD test of monthly comparison of sea surface temperature (SST) and chlorophyll a concentration (CHL). Bold values indicate no significant difference (p > 0.05). (DOCX) [file pone.0164440.s001.docx]

**Supplementary Material**

Macena and Hazin 2016 - Whale Shark Presence at the Archipelago of São Pedro and São Paulo, Brazil

S1 Table –Summary of TukeyHSD test results from the comparisons environmental variables per month.

|  | **SST** | | | |  | **CHL** | | | |
| --- | --- | --- | --- | --- | --- | --- | --- | --- | --- |
| **Month** | **diff** | **lwr** | **upr** | **p** |  | **diff** | **lwr** | **upr** | **p** |
| 2-1 | 0.3233 | 0.2353 | 0.4112 | 0.0000 |  | 0.0107 | -0.0008 | 0.0222 | **0.0958** |
| 3-1 | 0.6993 | 0.6115 | 0.7872 | 0.0000 |  | -0.0156 | -0.0256 | -0.0055 | 0.0000 |
| 4-1 | 1.0731 | 0.9852 | 1.1610 | 0.0000 |  | -0.0243 | -0.0341 | -0.0144 | 0.0000 |
| 5-1 | 1.3379 | 1.2500 | 1.4257 | 0.0000 |  | -0.0271 | -0.0368 | -0.0175 | 0.0000 |
| 6-1 | 0.2586 | 0.1707 | 0.3464 | 0.0000 |  | 0.0193 | 0.0097 | 0.0288 | 0.0000 |
| 7-1 | -0.5228 | -0.6107 | -0.4350 | 0.0000 |  | 0.0883 | 0.0788 | 0.0979 | 0.0000 |
| 8-1 | -0.8207 | -0.9086 | -0.7328 | 0.0000 |  | 0.0546 | 0.0450 | 0.0643 | 0.0000 |
| 9-1 | -0.6197 | -0.7075 | -0.5318 | 0.0000 |  | 0.0154 | 0.0057 | 0.0252 | 0.0000 |
| 10-1 | -0.1134 | -0.2012 | -0.0255 | 0.0015 |  | -0.0285 | -0.0382 | -0.0187 | 0.0000 |
| 11-1 | -0.1918 | -0.2796 | -0.1039 | 0.0000 |  | -0.0059 | -0.0157 | 0.0038 | **0.7023** |
| 12-1 | -0.1675 | -0.2554 | -0.0797 | 0.0000 |  | 0.0088 | -0.0014 | 0.0190 | **0.1770** |
| 3-2 | 0.3761 | 0.2881 | 0.4640 | 0.0000 |  | -0.0263 | -0.0368 | -0.0158 | 0.0000 |
| 4-2 | 0.7498 | 0.6619 | 0.8378 | 0.0000 |  | -0.0350 | -0.0453 | -0.0246 | 0.0000 |
| 5-2 | 1.0146 | 0.9267 | 1.1026 | 0.0000 |  | -0.0378 | -0.0479 | -0.0277 | 0.0000 |
| 6-2 | -0.0647 | -0.1526 | 0.0233 | **0.4035** |  | 0.0086 | -0.0015 | 0.0186 | **0.1841** |
| 7-2 | -0.8461 | -0.9340 | -0.7581 | 0.0000 |  | 0.0776 | 0.0676 | 0.0877 | 0.0000 |
| 8-2 | -1.1440 | -1.2319 | -1.0560 | 0.0000 |  | 0.0440 | 0.0338 | 0.0541 | 0.0000 |
| 9-2 | -0.9429 | -1.0309 | -0.8550 | 0.0000 |  | 0.0047 | -0.0055 | 0.0150 | **0.9384** |
| 10-2 | -0.4366 | -0.5246 | -0.3487 | 0.0000 |  | -0.0392 | -0.0494 | -0.0289 | 0.0000 |
| 11-2 | -0.5150 | -0.6030 | -0.4271 | 0.0000 |  | -0.0166 | -0.0269 | -0.0064 | 0.0000 |
| 12-2 | -0.4908 | -0.5787 | -0.4028 | 0.0000 |  | -0.0019 | -0.0126 | 0.0088 | **1.0000** |
| 4-3 | 0.3738 | 0.2859 | 0.4616 | 0.0000 |  | -0.0087 | -0.0174 | 0.0000 | 0.0510 |
| 5-3 | 0.6385 | 0.5507 | 0.7264 | 0.0000 |  | -0.0115 | -0.0200 | -0.0031 | 0.0005 |
| 6-3 | -0.4408 | -0.5286 | -0.3529 | 0.0000 |  | 0.0348 | 0.0265 | 0.0432 | 0.0000 |
| 7-3 | -1.2222 | -1.3100 | -1.1343 | 0.0000 |  | 0.1039 | 0.0955 | 0.1122 | 0.0000 |
| 8-3 | -1.5200 | -1.6079 | -1.4322 | 0.0000 |  | 0.0702 | 0.0618 | 0.0787 | 0.0000 |
| 9-3 | -1.3190 | -1.4068 | -1.2311 | 0.0000 |  | 0.0310 | 0.0224 | 0.0396 | 0.0000 |
| 10-3 | -0.8127 | -0.9006 | -0.7249 | 0.0000 |  | -0.0129 | -0.0215 | -0.0043 | 0.0001 |
| 11-3 | -0.8911 | -0.9789 | -0.8032 | 0.0000 |  | 0.0096 | 0.0010 | 0.0183 | 0.0139 |
| 12-3 | -0.8669 | -0.9547 | -0.7790 | 0.0000 |  | 0.0244 | 0.0152 | 0.0335 | 0.0000 |
| 5-4 | 0.2648 | 0.1769 | 0.3526 | 0.0000 |  | -0.0028 | -0.0111 | 0.0054 | **0.9935** |
| 6-4 | -0.8145 | -0.9024 | -0.7267 | 0.0000 |  | 0.0435 | 0.0354 | 0.0516 | 0.0000 |
| 7-4 | -1.5959 | -1.6838 | -1.5081 | 0.0000 |  | 0.1126 | 0.1045 | 0.1207 | 0.0000 |
| 8-4 | -1.8938 | -1.9817 | -1.8059 | 0.0000 |  | 0.0789 | 0.0707 | 0.0872 | 0.0000 |
| 9-4 | -1.6928 | -1.7806 | -1.6049 | 0.0000 |  | 0.0397 | 0.0313 | 0.0481 | 0.0000 |
| 10-4 | -1.1865 | -1.2743 | -1.0986 | 0.0000 |  | -0.0042 | -0.0126 | 0.0042 | **0.8941** |
| 11-4 | -1.2649 | -1.3527 | -1.1770 | 0.0000 |  | 0.0183 | 0.0099 | 0.0267 | 0.0000 |
| 12-4 | -1.2406 | -1.3285 | -1.1528 | 0.0000 |  | 0.0331 | 0.0241 | 0.0420 | 0.0000 |
| 6-5 | -1.0793 | -1.1671 | -0.9914 | 0.0000 |  | 0.0464 | 0.0385 | 0.0542 | 0.0000 |
| 7-5 | -1.8607 | -1.9486 | -1.7728 | 0.0000 |  | 0.1154 | 0.1076 | 0.1233 | 0.0000 |
| 8-5 | -2.1586 | -2.2464 | -2.0707 | 0.0000 |  | 0.0818 | 0.0738 | 0.0898 | 0.0000 |
| 9-5 | -1.9575 | -2.0454 | -1.8697 | 0.0000 |  | 0.0425 | 0.0344 | 0.0507 | 0.0000 |
| 10-5 | -1.4512 | -1.5391 | -1.3634 | 0.0000 |  | -0.0013 | -0.0095 | 0.0068 | **1.0000** |
| 11-5 | -1.5296 | -1.6175 | -1.4418 | 0.0000 |  | 0.0212 | 0.0130 | 0.0293 | 0.0000 |
| 12-5 | -1.5054 | -1.5932 | -1.4175 | 0.0000 |  | 0.0359 | 0.0272 | 0.0446 | 0.0000 |
| 7-6 | -0.7814 | -0.8693 | -0.6936 | 0.0000 |  | 0.0691 | 0.0613 | 0.0768 | 0.0000 |
| 8-6 | -1.0793 | -1.1671 | -0.9914 | 0.0000 |  | 0.0354 | 0.0275 | 0.0433 | 0.0000 |
| 9-6 | -0.8782 | -0.9661 | -0.7904 | 0.0000 |  | -0.0038 | -0.0119 | 0.0042 | **0.9239** |
| 10-6 | -0.3720 | -0.4598 | -0.2841 | 0.0000 |  | -0.0477 | -0.0557 | -0.0397 | 0.0000 |
| 11-6 | -0.4503 | -0.5382 | -0.3625 | 0.0000 |  | -0.0252 | -0.0332 | -0.0172 | 0.0000 |
| 12-6 | -0.4261 | -0.5140 | -0.3382 | 0.0000 |  | -0.0105 | -0.0191 | -0.0019 | 0.0041 |
| 8-7 | -0.2979 | -0.3857 | -0.2100 | 0.0000 |  | -0.0337 | -0.0416 | -0.0258 | 0.0000 |
| 9-7 | -0.0968 | -0.1847 | -0.0090 | 0.0166 |  | -0.0729 | -0.0809 | -0.0649 | 0.0000 |
| 10-7 | 0.4095 | 0.3216 | 0.4973 | 0.0000 |  | -0.1168 | -0.1248 | -0.1088 | 0.0000 |
| 11-7 | 0.3311 | 0.2432 | 0.4189 | 0.0000 |  | -0.0943 | -0.1023 | -0.0862 | 0.0000 |
| 12-7 | 0.3553 | 0.2675 | 0.4432 | 0.0000 |  | -0.0795 | -0.0881 | -0.0709 | 0.0000 |
| 9-8 | 0.2010 | 0.1132 | 0.2889 | 0.0000 |  | -0.0392 | -0.0474 | -0.0311 | 0.0000 |
| 10-8 | 0.7073 | 0.6195 | 0.7952 | 0.0000 |  | -0.0831 | -0.0912 | -0.0750 | 0.0000 |
| 11-8 | 0.6289 | 0.5411 | 0.7168 | 0.0000 |  | -0.0606 | -0.0688 | -0.0524 | 0.0000 |
| 12-8 | 0.6532 | 0.5653 | 0.7410 | 0.0000 |  | -0.0458 | -0.0546 | -0.0371 | 0.0000 |
| 10-9 | 0.5063 | 0.4184 | 0.5941 | 0.0000 |  | -0.0439 | -0.0522 | -0.0356 | 0.0000 |
| 11-9 | 0.4279 | 0.3400 | 0.5158 | 0.0000 |  | -0.0214 | -0.0297 | -0.0131 | 0.0000 |
| 12-9 | 0.4521 | 0.3643 | 0.5400 | 0.0000 |  | -0.0066 | -0.0155 | 0.0022 | **0.3763** |
| 11-10 | -0.0784 | -0.1662 | 0.0095 | **0.1351** |  | 0.0225 | 0.0142 | 0.0308 | 0.0000 |
| 12-10 | -0.0541 | -0.1420 | 0.0337 | **0.6836** |  | 0.0373 | 0.0284 | 0.0461 | 0.0000 |
| 12-11 | 0.0242 | -0.0636 | 0.1121 | **0.9991** |  | 0.0147 | 0.0059 | 0.0236 | 0.0000 |

Output from TukeyHSD test of monthly comparison of sea surface temperature (SST) and chlorophyll a concentration (CHL). Bold values indicate no significant difference (p > 0.05).
